# Characterization of Frequently Mutated Cancer Genes and Tumor Mutation Burden in Chinese Breast Cancer

**DOI:** 10.3389/fonc.2021.618767

**Published:** 2021-04-21

**Authors:** Weikai Xiao, Guochun Zhang, Bo Chen, Xiaoqing Chen, Lingzhu Wen, Jianguo Lai, Xuerui Li, Min Li, Hao Liu, Jing Liu, Han Han-Zhang, Analyn Lizaso, Ning Liao

**Affiliations:** ^1^Department of Breast Cancer, Cancer Center, Guangdong Provincial People's Hospital, Guangdong Academy of Medical Sciences, Guangzhou, China; ^2^Department of Breast, Foshan Women and Children Hospital, Foshan, China; ^3^Burning Rock Biotech, Guangzhou, China

**Keywords:** genomic alteration, breast cancer, clinical impact, tumor mutational burden, gene fusion

## Abstract

**Objectives:**

Various genomic alterations and genomic signatures, including *ERBB2* amplification, mutations in *PIK3CA*, *AKT1*, and *ESR1*, and tumor mutational burden (TMB), have become important biomarkers for treatment selection in breast cancer (BC). This study aimed to investigate the mutational features of Chinese early-stage BC patients.

**Methods:**

Tumors and matched blood samples collected from 589 Chinese patients with early-stage BC were sequenced using a commercial gene panel consisting of 520 cancer-related genes to analyze all types of genomic alterations and estimate the TMB status.

**Results:**

A total of 18 genes were found to be more frequently mutated (*P*<0.05) or amplified (*P*<0.05) in stage T3–4 tumors as compared with T1–2 tumors. A total of 18 genes were found to be differentially mutated (*P*<0.05) or amplified (*P*<0.05) in patients with lymph node metastasis than those without lymph node metastasis. Younger patients (≤35 years) were more frequently identified with mutations or gene amplifications in eleven genes (*P*<0.05). TMB >10mutations/Mb were found in 5.7% of our cohort. Although the TMB was similar for various molecular subtypes between our cohort and the BC cohort of The Cancer Genome Atlas (TCGA) study, the TMB were statistically different for HR+/HER-, HR+/HER2+, and triple-negative subtypes between our cohort and African Americans in the TCGA study. As compared to the TCGA BC cohort, our cohort had a much earlier median age of diagnosis (48 vs. 58 years, *P*<0.001), and had significantly lower frequency of triple-negative subtype (11.5% vs. 18.4%, *P*<0.001) and invasive lobular BC (2.4% vs. 19.0%, *P*<0.001). Further subgroup analyses revealed that mutation rates in various genes including *TP53*, *ERBB2*, and *PIK3CA* were distinct for patients who were younger (≤35 years), had triple-negative or invasive lobular BC in our cohort than in the TCGA cohort.

**Conclusions:**

This study revealed distinct mutational features of various molecular subtypes of early-stage BC among Chinese patients. Moreover, we provide new insights into the differences in early-stage BC between the East and West.

## Introduction

Breast cancer is the most common cancer and the leading cause of cancer deaths in women globally and China (1, 2). According to molecular subtype, breast cancer is divided into Luminal A, Luminal B, HER2-enriched, and triple-negative subtypes, among which the luminal subtype, accounting for 65% to 70% of the cases, has the highest proportion (3). In recent years, next-generation sequencing (NGS) has accelerated the systematic characterization of the genomic landscape of breast cancer, which contributed to our current understanding of the unique and shared genomic features of the four breast cancer molecular subtypes (4–7). Genomic aberrations in a number of genes, including *TP53* and *PIK3CA*, have been implicated in the development of breast cancer (7). While genomic studies of breast cancer are mainly performed in patients from Western countries (5, 8, 9), the increasing efforts in investigating the features of Chinese patients with breast cancer have revealed their unique epidemiological characteristics. As compared to Caucasians, the Chinese patients have an earlier age of onset for breast cancer with peak incidence observed between 45 and 55 years as well as a distinct molecular subtype distribution with a higher proportion of luminal and lower proportion of triple-negative subtypes (9). The incidence of breast cancer in China has been rising gradually and may eventually surpass that of Western countries (10). To better understand the etiological differences between breast cancer in China and Western countries, we retrospectively analyzed the genomic data of 589 Chinese patients with various molecular and histological subtypes of early-stage (stage I–III) breast cancer to elucidate their mutational landscape. Furthermore, we also performed subgroup analysis to compare the genomic data of a subgroup from our cohort to the breast cancer dataset from the Cancer Genome Atlas (TCGA) with similar clinicopathological features to identify distinct mutational features in our population.

## Methods

### Patients

Chinese patients diagnosed with various histology of early-stage (stage I-III) breast cancer in Guangdong Provincial People's Hospital (GDPH) who submitted paired breast tissue samples and blood samples for targeted sequencing to Burning Rock Biotech, a clinical laboratory accredited by the College of American Pathologist (CAP) and certified by the Clinical Laboratory Improvement Amendments (CLIA), were included in this retrospective study. Hormone receptor (HR) (*i.e.*, estrogen receptor [ER] and progesterone receptor [PR]) and human epidermal growth factor receptor 2 (HER2) status were defined according to the guidelines of the American Society of Clinical Oncology (ASCO) and CAP.

### DNA Isolation and Capture-Based Targeted DNA Sequencing

As described previously (11, 12), tissue DNA was extracted from formalin-fixed, paraffin-embedded (FFPE) tumor tissues using QIAamp DNA FFPE tissue kit (Qiagen, Hilden, Germany). Genomic DNA was extracted from blood samples using QIAamp DNA blood mini kit (Qiagen, Hilden, Germany). A minimum of 50 ng of DNA is required for NGS library construction. Tissue DNA was sheared using Covaris M220 (Covaris, MA, USA), followed by end repair, phosphorylation, and adaptor ligation. Fragments between 200 and 400 bp were purified (Agencourt AMPure XP Kit, Beckman Coulter, CA, USA), followed by hybridization with capture probes baits, hybrid selection with magnetic beads, and PCR amplification. The quality and the size of the fragments were assessed with the high sensitivity DNA kit using Bioanalyzer 2100 (Agilent Technologies, CA, USA). Target capture was performed using a commercial panel consisting of 520 cancer-related genes, spanning 1.64 megabases (Mb) of the human genome (OncoScreen Plus, Burning Rock Biotech, Guangzhou, China). The genes included in the panel are listed in **Supplementary Table S1**. Indexed samples were sequenced on Nextseq500 (Illumina, Inc., USA) with paired-end reads and average sequencing depth of 1,000× for tissue samples and 10,000× for blood samples.

### Sequence Data Analysis

Sequence data were analyzed using optimized bioinformatics pipelines for somatic and germline variant calling and annotation (11, 12) . Briefly, the sequence data were mapped to the reference human genome (hg19) using Burrows-Wheeler Aligner v.0.7.10 (13). Local alignment optimization, duplication marking and variant calling were performed using Genome Analysis Tool Kit v.3.2 (14), and VarScan v.2.4.3 (15). Sequencing data from tissue samples were compared against their own white blood cell control to identify somatic variants. Variants were filtered using the VarScan fpfilter pipeline, loci with depth less than 100 were filtered out. Base calling in plasma and tissue samples required at least eight supporting reads for single nucleotide variations (SNV) and two and five supporting reads for insertion-deletion variations (indel), respectively. Variants with population frequency over 0.1% in the ExAC, 1,000 Genomes, dbSNP or ESP6500SI-V2 databases were grouped as single nucleotide polymorphisms and excluded from further analysis. The remaining variants were annotated with ANNOVAR (2016-02-01 release) (16) and SnpEff v.3.6 (17). Analysis of DNA translocation was performed using Factera v.1.4.3 (18). Copy number variations (CNV) were analyzed based on the depth of coverage data of capture intervals. Coverage data were corrected against sequencing bias resulting from GC content and probe design. The average coverage of all captured regions was used to normalize the coverage of different samples to comparable scales. Copy number (CN) was calculated based on the ratio between the depth of coverage in tumor samples and average coverage of an adequate number (n > 50) of samples without copy number variations as references per capture interval. CNV is called if the coverage data of the gene region was quantitatively and statistically significant from its reference control. The limit of detection for CNV is 1.5 for CN deletion and 2.64 for CN amplifications.

Tumor mutation burden per patient was computed as a ratio between the total number of mutations detected and the total coding region size of the panel used using the formula below. CNVs, fusions, large genomic rearrangements, and mutations occurring on the kinase domain of *EGFR* and *ALK* were excluded from the mutation count; hence, the total size of the coding region of the panel for estimating tumor mutation burden is 1.26 Mb for the 520-gene OncoScreen Plus panel.

$$Tumor\;mutation\;burden=\frac{mutation\;count\;(except\;for\;CNVs\;\;and\;fusion)}{total\;size\;of\;coding\;region\;of\;the\;panel\;used}$$

### Statistical Analysis

Categorical variables were summarized as frequency or percentage and compared using Fisher's exact test or chi-square test. The statistical test was two-sided, and P < 0.05 was considered statistically significant. The P values were corrected using the error discovery rate (FDR or Q value) and multiple hypothesis testing was performed according to the Benjamin-Hochberg program.

## Results

### Patient Characteristics

The clinical and pathological characteristics of the 589 patients included in our study are shown in **Supplementary Table S2**. The median age of the patients was 48 years. A majority (55.9%) of the patients were stage II at the time of diagnosis, followed by stage I with 23.3%, and stage III with 20.9%. The distribution of molecular subtypes were 73.3% HR+/HER2− subtype, 11.5% triple-negative, and 29.7% HER2-enriched subtypes.

### Mutational Profile

**Figure 1:**
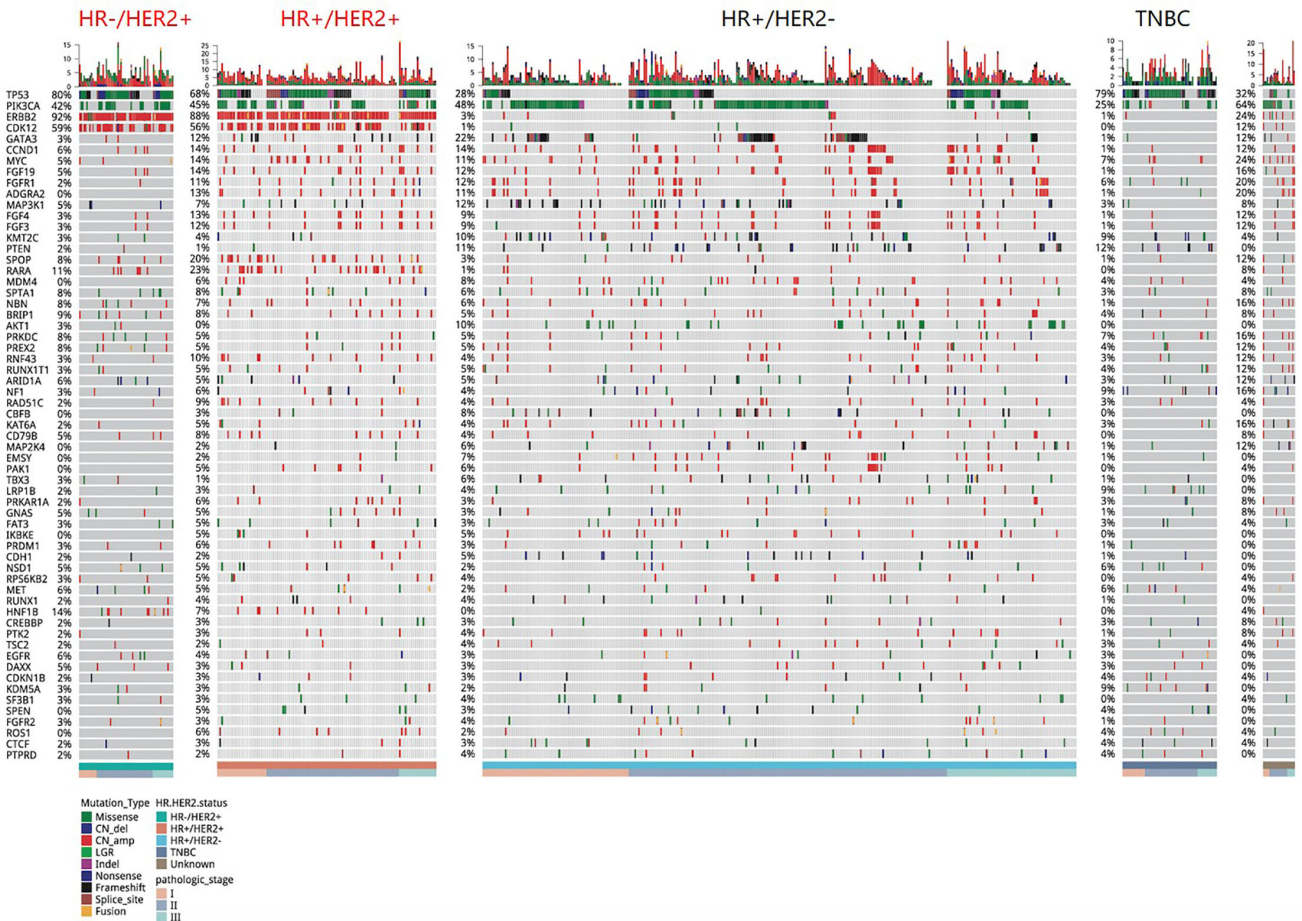
A summary of the genomic characteristics of 589 Chinese breast cancer patients. Oncoprint showed genetic changes with an incidence of more than 2%. According to the HR and HER2 status, the tumor samples were stratified into: HR+/HER2 (n = 321), HR+/HER2+ (n = 111), HR−/HER2+ (n = 64), triple-negative (n = 68), and unknown (n = 25). The sidebar summarizes the percentage of tumors with mutations in each gene. Different colors indicate different types of mutations. Clinicopathological features were annotated at the bottom. Indel, insertion or deletion variations; CN_amp, copy number amplification; CN_del, copy number deletion.

Targeted sequencing was performed on tissue samples of all 589 patients. A total of 5,592 somatic mutations in 418 genes were identified from 577 patients, resulting in a mutation detection rate of 98.0% (577/589). Various mutation types were detected including 2,109 CN amplification, 1,780 missense mutations, 389 frameshift mutations, 214 nonsense mutations, 183 splice-site variants, 81 indels, 78 fusions, and 24 CN deletions. **Figure 1** summarized the mutation landscape of Chinese early-stage breast cancer. *TP53* (47.0%), *PIK3CA* (45.0%), and *ERBB2* (30.0%) were the most frequently mutated genes in our cohort. Other genes that were mutated in ≥10% of the patients included *CDK12* (18.0%), *GATA3* (15.0%), *CCND1* (12.0%), *FGF19* (11.0%), and *FGFR1* (10.0%).

**Figure 2:**
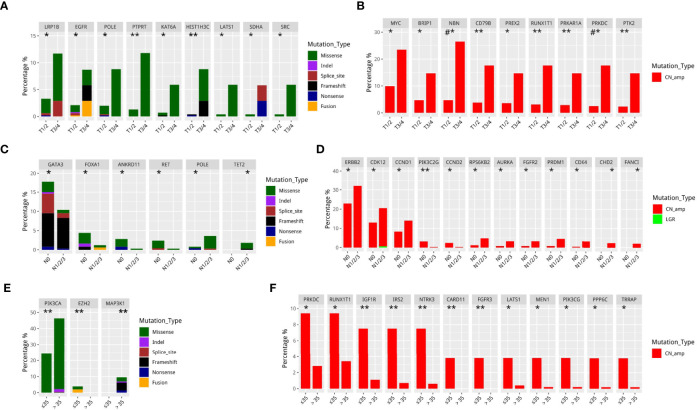
Differentially mutated genes in different Tumor stage, Nodal stage, and younger breast cancer in GDPH cohort. **(A, B)** Differentially mutated (SNV/Indel/fusion) **(A)** and amplified **(B)** genes among T1–2 and T3–4 breast cancer. **(C, D)** Differentially mutated (SNV/Indel/fusion) **(C)** and amplified **(D)** genes among patients based on the presence and absence of lymph node metastasis. **(E, F)** Differentially mutated (SNV/Indel/fusion) **(E)** and amplified **(F)** genes between younger (≤35 years) and older (>35 years) patients with breast cancer. *P<0.05; **P≤0.01; ^#^*P*≤0.001.

We then performed further analysis to understand the distinct mutational features of our cohort across various clinical features including tumor status (T), lymph node status (N), and age at diagnosis. As compared to T1–2 tumors, T3–4 tumors had significantly more mutations in nine genes including *LPR1B*, *EGFR*, *POLE*, *PTPRT*, *KAT6A*, *HIST1H3C*, *LATS1*, *SDHA*, and *SRC* (*P*<0.05, **Figure 2A**) and significantly more CN amplifications in nine genes including *MYC*, *BRIP1*, *NBN*, *CD79B*, *PREX2*, *RUNX1T1*, *PRKAR1A*, *PRKDC*, and *PTK2* (*P*<0.05, **Figure 2B**). As compared to patients without lymph node metastasis, patients with lymph node metastasis were found to harbor significantly more mutations in two genes, *POLE* and *TET2* (*P*<0.05, **Figure 2C**) and significantly more CN amplifications in 10 genes including *ERBB2*, *CDK12*, *CCND1*, *RPS6KB2*, *AURKA*, *FGFR2*, *PRDM1*, *CDK4*, *CHD2*, and *FANCI* (*P* <0.05, **Figure 2D**). In contrast, patients without lymph node metastasis were found to have more frequent mutation in four genes including *GATA3*, *FOXA1*, *ANKRD11*, and *RET* (*P*<0.05, **Figure 2C**) and more CN amplifications in two genes including *PIK3C2G* and *CCND2* (*P*<0.05, **Figure 2D****)**. Younger patients (≤35 years) with breast cancer had more mutations in *EZH2* but fewer mutations in *PIK3CA* and *MAP3K1* (*P*<0.05, **Figure 2E**) and more CN amplifications in 12 genes including *PRKDC*, *RUNX1T1*, *IGF1R*, *IRS2*, *NTRK3*, *CARD11*, *FGFR3*, *LATS1*, *MEN1*, *PIK3CG*, *PPP6C*, and *TRRAP* (*P*<0.05, **Figure 2F**).

### *ERBB2* Mutations

In addition to the mutational landscape of the cohort, we also analyzed the genetic features of our cohort in specific genes. A total of 205 *ERBB2* aberrations were detected in 175 of the 589 breast cancer cases, revealing an *ERBB2* mutation rate of 29.7%. Various mutation types detected from our cohort included CN amplification (n=166), missense mutations (n=14), fusion (n=12), synonymous mutations (n=7), splice-site mutations (n=3), and intronic mutations (n=3). *ERBB2* amplifications were detected in 28.2% (166/589) of the cohort; of them, eight patients had HER2-negative subtype and may be candidates for targeted HER2 therapy. We also found that *ERBB2* aberrations were more likely to co-occur with *CDK12* (Odds ratio (OR)=10), *RAPA* (OR=10), and *SPOP* (OR=7.4). Moreover, mutual exclusivity were found between *ERBB2* aberrations and *GATA3* (OR=0.5), *KMT2C* (OR=0.3), *PTEN* (OR=0.2), *AKT1* (OR=0.2), *CBFB* (OR=0.2), or *MAP2K4* (OR=0.1). In addition, 12 novel *ERBB2* gene fusion partners were detected in seven patients from our cohort, including *KSR1-ERBB2*, *KRTAP1-4-ERBB2*, *PIP4K2B-ERBB2*, *MED1-ERBB2*, *METRNL-ERBB2*, *SRCIN1-ERBB2*, *GLRA3-ERBB2*, *LOC100288778-ERBB2*, *IKZF3-ERBB2*, *ABCA9-ERBB2*, *PPP1R1B-ERBB2*, and *ABCA6-ERBB2*.

### *ESR1* Mutations

A total of 2.7% (16/589) of patients were detected with *ESR1* mutations, including six CN amplifications, six missense mutations, two frameshift mutations, one splice-site mutation, and one synonymous mutation. A majority (68.8%; 11/16) of the *ESR1* mutation were detected from stage III breast cancer. Interestingly, 25.0% (4/16) of the *ESR1* mutation-positive patients were hormone receptor-negative. Moreover, all breast cancers with *ESR1* mutations were invasive ductal carcinomas, with a majority of luminal B subtype (75.0%, 12/16) and the remaining four patients had HER2-enriched (n=2) and triple-negative (n=2) subtypes.

### Somatic Alterations in Other Oncogenic Drivers

In addition to *ERBB2* and *ESR1*, we also analyzed the mutation frequency of other oncogenic drivers, including *KRAS, ROS1, ALK, MET, NTRK*, and *EGFR*, to identify potentially actionable targets. *KRAS* mutations were detected in 2.7% (16/589) of the cohort, most of which were CN amplification (87.5%, 14/16). *ROS1* mutations were detected in 4.1% (24/589) of the cohort; of which, two (8.33%) patients were detected with novel *ROS1* gene fusions including *CNTN3-ROS1*, *HACE1-ROS1*, and *NUS1-ROS1*. *ALK* mutations were detected in 2.0% (12/589) of the cohort; of which, three patients were detected with *ALK* gene fusions including *ADGRL3-AS1-ALK*, *EML4-ALK*, and *STRN-ALK*. Gene mutations in *NTRK1/2/3* were detected in 4.9% (29/589) of patients, with a majority (62.1%, 18/29) of CN amplifications. No patient was detected with *NTRK* gene fusion in our cohort. Gene mutations in *MET* were detected in 4.9% (28/589) of the cohort; of which, three patients were detected with novel *MET* gene fusions including *AGBL3-MET*, *MMD-MET*, *LVCAT5-MET*, and *XKR6-MET*.

### Immunotherapy-Related Markers

In addition to gene mutations related to targeted therapy, we also investigated gene mutations and TMB status that are associated with immunotherapy response. Gene alterations in *CD274/PD-L1* were detected in 1.4% (8/589) of the cohort, six of which were CN amplification. Among these eight cases, only one case was triple-negative subtype, six patients had luminal B, and one patient had HER2-enriched subtype. *BRAF* alterations were detected in 1.5% (9/589) of the cohort, including four missense mutations, three CN amplifications, and two intronic mutations. The mutation frequencies of *POLE* and *POLD* genes were 3.4% (20/589) and 2.2% (13/589), respectively. Mutations in both genes had been reported by previous studies to predict the efficacy of immunotherapy.

The median TMB of our cohort was four (range, 0–46) mutations/Mb. Only 5.7% of the cohort had TMB greater than 10 mutations/Mb.

**Figure 3:**
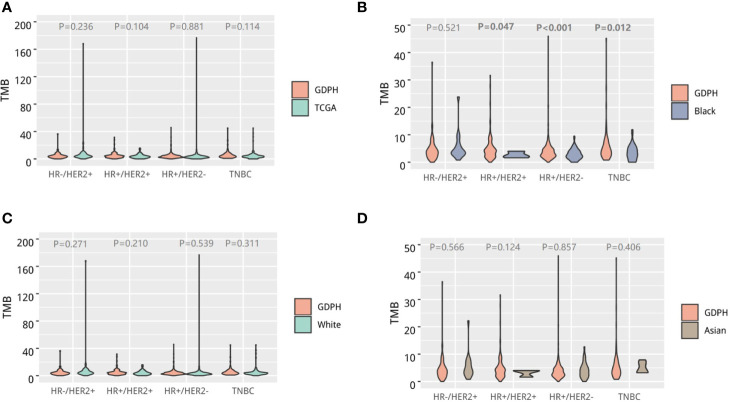
Tumor mutational burden (TMB) of Chinese breast cancer. Violin diagram comparing the distribution of TMB across molecular subtypes between Chinese breast cancer and the whole TCGA breast cancer cohort **(A)**, African-American patients in the TCGA cohort **(B)**, Caucasian patients in the TCGA cohort **(C)**, and Asian patients in the TCGA cohort **(D)**. The outline of the violins shows the mirrored kernel density, with the red dot indicating the median. Statistical comparisons were performed using Mann–Whitney *U* tests.

Subsequently, we compared the TMB of our cohort and the breast cancer cohort of the TCGA study. In general, no difference in TMB was observed across molecular subtypes between our cohort and the TCGA breast cancer cohort (**Figure 3A****)**. We then conducted further subgroup analysis based on ethnicity and found that the TMB of HR+/HER-, HR+/HER2+, and triple-negative subtypes in our cohort were statistically different from those of the African Americans (black) from the TCGA study (**Figure 3B****)**. However, no statistical difference was observed in TMB between our cohort and the Caucasians (white; **Figure 3C**) and Asians from the TCGA study (**Figure 3D**).

### PI3K/AKT/mTOR Pathway, Cell Cycle Pathway, and FGFR Pathway

We further analyzed the pathways most commonly mutated in our cohort. Among the genes involved in the PI3K-AKT pathway, the most commonly mutated were *PIK3CA* (45%), *PTEN* (7.5%), and *AKT1* (5.9%). Among the cell cycle pathway-related genes, the most commonly mutated were *CCND1* (45%), *MYC* (11%), and *CDKN1B* (3.0%). Among the genes involved in the FGFR pathway, *FGF19* and *FGFR1* were the most frequently mutated, with an incidence of 11% and 10%, respectively. The other FGFR pathway-related genes detected from our cohort were *FGF4* (n=48), *FGF3* (n=48), *FGFR2* (n=18), *FGFR4* (n=11), *FGFR3* (n=5), *FGF14* (n=7), *FGF12* (n=2), and *FGF7* (n=1).

### Homologous Recombination Repair Pathway

**Figure 4:**
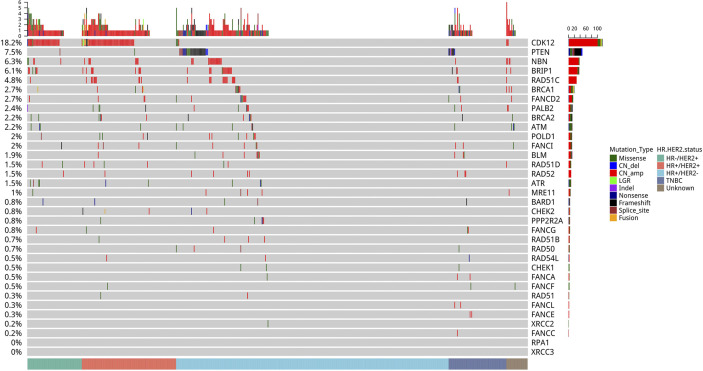
Mutation profile of genes in the homologous recombination repair (HRR) pathway. Tumor samples were grouped by molecular subtype: HR+/HER2− (n = 321), HR+/HER2+ (n = 111), HER2-enriched (n = 64), and triple-negative breast cancer (TNBC) (n = 68) as indicated by the annotation at the bottom. The mutation frequency for each gene is shown on the left. Colors indicate the mutation types.

Among the homologous recombination repair (HRR) pathway-related genes, *CDK12* (18.2%) was the most frequently mutated, followed by *PTEN* (7.5%), *NBN* (6.3%), *BRIP1* (4.3%), *RAD51C* (4.8%), *BRCA1* (2.7%), *FANCD2* (2.7%), *PALB2* (2.4%), *BRCA2* (2.2%), *ATM* (2.2%), *POLD1* (2.0%), and *FANCI* (2.0%) (**Figure 4**).

We further explored the pathogenic/likely pathogenic (P/LP) germline mutations in our cohort. P/LP germline mutations were detected from 11.2% (66/589) of our cohort. P/LP germline mutations were detected in genes including *BRCA2* (n=19), *BRCA1* (n=15), *ATM* (n=4), *BARD1* (n=1), *BRIP1* (n=2), *CDH1* (n=3), *CHEK1* (n=1), *FANCA* (n=3), *FANCL* (n=1), *MUTYH* (n=4), *PALB2* (n=6), *PMS2* (n=1), *PTEN* (n=1), *RAD51C* (n=2), *SDHA* (n=1), and *TP53* (n=2). Germline *BRCA1/2* mutations were detected in 34 patients (5.8%). Other germline mutations in genes related to the HRR pathway were detected in 21 patients (3.6%). According to molecular subtypes, P/LP germline mutations were detected in 39 patients with HR+/HER2−, 10 patients with HR+/HER2+, six patients with HR-/HER2+, and 10 patients with triple-negative subtype. *BRCA1* mutations were detected in 15 patients; of which, six patients had HR +/HER2−, one had HR+/HER2+, three had HR-/HER2+, and five had triple-negative subtypes. *BRCA2* mutations were detected in 19 patients; of which, 15 patients had HR+/HER2−, two had HR+/HER2+, one had HR-/HER2+, and one had unknown subtype. No germline *BRCA2* P/LP mutation was detected in triple-negative subtype.

### Concurrent and Mutually Exclusive Genomic Aberrations

**Figure 5:**
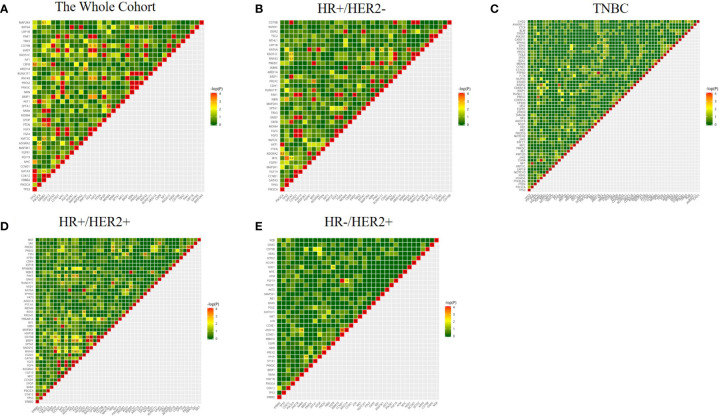
Coexisting and mutually exclusive gene mutations in 520 cancer-related gene aberrations in the whole cohort **(A)** and various molecular subtypes of breast cancer **(B–E)**. Genes with a mutation rate of ≥4% were included in the analysis; the color represents the trend of correlation, the numbers represent the mutually exclusive association of genes, Odds ratio (OR) >5 tends to be associated, OR<0.5 tends to be mutually exclusive.

To understand the relationship between genomic aberrations, we further analyzed which somatic gene mutations were likely to exist together or in mutual exclusivity in the whole cohort (**Figure 5A**) as well as each molecular subtype of breast cancer (**Figures 5B–E**). In the entire cohort (**Figure 5A**), *TP53* mutations were more likely to have a mutually exclusive relationship with mutations in *GATA3* (OR=0.1), *AKT1* (OR=0.3), and *CBFB* (OR=0.2). *PIK3CA* mutations were more likely to have a mutually exclusive relationship with mutations in *ADGRA2* (OR=0.4) and *AKT1* (OR=0.1). *ERBB2* mutations were more likely to co-exist with mutations in *CDK12* (OR=10), *RAPA* (OR=10), and *SPOP* (OR=7.4), but were mutually exclusive with mutations in *GATA3* (OR=0.5), *PTEN* (OR=0.3), *AKT1* (OR=0.2), *CBFB* (OR=0.2), and *MAP2K4* (OR=0.1). Being located in the same chromosome (chromosome 11q13), *CCND1* amplifications were more likely to be co-amplified with *FGF3* (OR=10), *FGF4* (OR=10), *FGF19* (OR=10), *EMSY* (OR=10), and *PAK1* (OR=10). *GATA3* mutations were more likely to co-occur with mutations in *BRIP1* (OR=5.7), *CBFB* (OR=10), and *CD79B* (OR=10).

### Comparison of Mutation Frequency Between Chinese and TCGA Breast Cancer Cohorts

To understand the distinct clinical characteristics of breast cancer among Chinese patients, we compared our data with the breast cancer cohort of the TCGA study. Compared to the TCGA breast cancer cohort, our cohort had a much younger median age of diagnosis (48 years for GDPH vs. 58 years for TCGA, *P*<0.001), and had significantly lower frequency of triple-negative breast cancer (11.5% of GDPH vs. 18.4% of TCGA, *P*<0.001) and invasive lobular breast cancer (2.4% of GDPH vs. 19.0% of TCGA, *P*<0.001). These results indicate the distinct clinicopathological characteristics of breast cancer between Chinese and Western breast cancer.

We then performed subgroup analysis of the mutation profile of patients in our cohort and those in the TCGA cohort with similar clinical characteristics, particularly in age (*i.e.* ≤35 years), breast cancer histological subtype (*i.e.* invasive lobular breast cancer), and molecular subtype (*i.e.* triple-negative subtype).

As compared with the TCGA cohort, younger breast cancer patients (≤35 years) in our cohort had higher mutation rates in *TP53* (51% vs. 30%) and *ERBB2* (34% vs. 24%), and lower mutation rates in *PIK3CA* (25% vs. 30%) and *GATA3* (15% vs. 24%). Meanwhile, the TCGA cohort had more mutations in *RPTOR*, *PRKAR1A*, and *RAD51C* than our cohort (**Supplementary Figure S1**).

We also compared the mutation profile of patients with triple-negative breast cancer in our cohort and those in the TCGA cohort. Our cohort had a higher mutation rate in *PIK3CA* (25% vs 14%) and lower mutation rate in *TP53* (79% vs 90%) and *PTEN* (12% vs 22%) than the TCGA cohort. *KRAS* gene mutations were detected in 9% of our cohort but were relatively rare (5.4%) in the TCGA cohort (**Supplementary Figure S2**).

Lastly, we compared the mutation profile of patients with invasive lobular breast cancer in our cohort and those in the TCGA cohort. Our cohort had higher mutation rates in *CDH1* (86% vs 54%) and *PIK3CA* (79% vs 37%) than in the TCGA cohort. However, the TCGA cohort had more frequent mutations in genes including *CCND1* (19%), *FGF19* (19%), *FGF4* (19%), *FGF3* (18%), *IKBKE* (14%), *AKT3* (13%), *FH* (12%), *PTEN* (12%), *CDC73* (12%), *ABL2* (12%), *NOTCH2* (11%), *ERBB2* (11%), *NTRK1* (10%), *DDR2* (10%), and *FGFR1* (10%) than our cohort (**Supplementary Figure S3**).

Taken together, these data indicate that the mutational feature of Chinese breast cancer is distinct from the TCGA breast cancer cohort, particularly among younger patients, and patients with triple-negative subtype and invasive lobular breast cancer.

## Discussion

The main purpose of precision cancer medicine is to develop management strategies based on specific molecular events related to tumor progression. The use of targeted NGS sequencing increases the possibility of the application of targeted therapies for breast cancer patients by enabling the detection of clinically-relevant genomic changes. In our cohort, 98.0% (577/589) of the samples showed at least one mutation across 520 cancer-related genes. *TP53* (47%), *PIK3CA* (45%), and *ERBB2* (30%) were mutated in more than 30% of the cohort. These three genes were also differentially mutated across molecular subtypes wherein *PIK3CA* mutations (48%) were mostly identified among HR+/HER2− tumors, *TP53* mutations (79%) were mostly identified among triple-negative subtype, and *ERBB2* mutations (88% in HR+/HER2+ and 92% in HR+/HER2−) were mostly identified among the HER2-positive subtype. In addition, we have discovered a number of candidate markers that have generally low incidence in breast cancer but have potential therapeutic value, including genetic aberrations in *KRAS*, *ROS1*, *ALK*, *MET*, *NTRK*, and *EGFR*. Certain mutations in these genes are associated with response to targeted therapy in other cancer types, but their therapeutic value in breast cancer requires further evaluation. These observations also suggest that comprehensive genomic analysis of breast cancer is required to facilitate the identification of the subset of patients who may benefit from targeted therapy.

*ERBB2* amplification is a predictive biomarker of response for HER2 targeted therapy in breast cancer. Conventional methods including immunohistochemistry and fluorescence *in situ* hybridization are still routinely used as clinical testing methods for HER2 overexpression and amplification; while the clinical value of panel-based NGS in breast cancer remains unclear. Through the use of NGS, we found that in addition to *ERBB2/HER2* amplification, genomic aberrations in *ERBB2* also exist as point mutations and gene fusions, albeit in much lower frequency than CN amplifications. In our cohort, we found that the common hotspot mutations in *ERBB2* were L755S, D769Y, and V777L, which are all located in the ERBB2 kinase domain and were shown to be activating mutations in various solid cancers (19). These three activating mutations were commonly detected from HER2 amplification negative tumors, with *ERBB2* L755S associated with HER2 inhibitor resistance (20). Since these mutations are missense mutations that can only be detected by DNA-based analysis using NGS or digital PCR, they may be missed by the conventional methods of HER2 assays. The *ESR1* mutation status of breast cancer is associated with resistance to endocrine therapy (21). In our cohort, 2.7% (16/589) of the patients have *ESR1* mutations, with 69% (11/16) of the patients harboring *ESR1* mutation had stage III breast cancer.

Furthermore, the subgroup comparative analysis revealed the distinct mutation landscape between our cohort and the TCGA cohort, particularly among younger patients, triple-negative breast cancer, and invasive lobular breast cancer, which may provide new insights into the differences between Chinese and Western breast cancers and may shed light on novel therapeutic targets.

The proportion of younger patients in our cohort is significantly higher than that in the TGCA cohort. To understand this difference at the molecular level, subgroup comparative analysis of the mutational features in both cohorts revealed higher mutation rates in *TP53*, *ERBB2*, and *CCND1* and lower mutation rates in *PIK3CA* and *GATA3* in our cohort than the TCGA cohort. As a tumor suppressor gene, mutations in *TP53* are closely related to the proliferation, invasion, and angiogenesis of cancer cells. *TP53* mutations were also implicated in the poor prognosis of breast cancer patients (22–24).

In both our cohort and the TCGA cohort, *CDH1* and *PIK3CA* were the most frequently mutated genes in invasive lobular breast cancers. Consistent with our findings, Desmedt et al. revealed that *CDH1* and *PIK3CA* were the major driver genes of invasive lobular breast cancer (25). However, the incidence of invasive lobular breast cancer in China was significantly lower than in the TCGA cohort. Our cohort had a higher mutation rate in *KMT2C* and fewer co-amplification of *CCND1/FGF3/4/19* than in the TCGA cohort. As an important epigenetic regulator, histone lysine methyltransferase 2C (KMT2C) is frequently mutated in a variety of human cancers and is considered to be essential in the development of many cancers (26). Rampias et al. found that the down-regulation of *KMT2C* in bladder cancer cells leads to extensive changes in the epigenetic status, DNA damage response, and expression of genes related to DNA repair (27). *KMT2C* mutation in diffuse gastric adenocarcinoma promotes epithelial to mesenchymal transition and is significantly associated with poor prognosis (28). It has been previously reported that *FGF19* is amplified in several cancer types and encodes a key autocrine signal known to promote tumorigenic growth (29). *FGF3* and *FGF4* genes are located in adjacent regions, and are within 0.2 Mb to the *FGF19* and *CCND1* genes in the chromosome 11q13 region. The amplification of chromosome 11q13 region is often observed in estrogen receptor-positive breast cancers and is associated with poor prognosis and treatment failure (30–32).

The mutation profiles we have observed from our cohort are similar to those reported in other studies. We have observed a higher mutation rate for *PIK3CA* and a lower mutation rate for *PTEN* for our cohort as compared to the TCGA breast cancer dataset. This observation is consistent with a recent report by Shao et al. (33). The amplification rate for *MYC*, *NOTCH2*, and *PTK2* was higher among the triple-negative subtype in the TCGA cohort than in our cohort. Previous studies have demonstrated that triple-negative breast cancer had a higher proportion of *MYC* gene amplification and increased mRNA expression than other breast cancer molecular subtypes (34). *NOTCH3* and *NOTCH2* mutations were detected in a subset of Chinese patients with triple-negative breast cancer. Meanwhile, *NOTCH2* amplification was observed in the triple-negative subgroup of the TCGA cohort. These observations suggest that the NOTCH signaling pathway is genetically altered and might be activated in a portion of triple-negative breast cancer, which is consistent with previous reports (35, 36). In addition, we observed higher *KRAS* mutations among the patients with triple-negative breast cancer in our cohort than in the TCGA cohort. Tokumaru et al. reported that *KRAS* signal-driven triple-negative breast cancer is associated with a good tumor immune microenvironment and better survival rate (37).

This study has some limitations. Our study only investigated the mutation landscape in 520 cancer-related genes, which might miss some novel regulators of breast cancer development. Although we have found distinct features between our cohort and the TCGA cohort, the sample size in certain subgroups limited our analysis. In addition, treatment and survival outcomes were not included in our analysis. We plan to reanalyze our data when either the disease-free survival or overall survival data is more mature, so we could determine the prognostic value of frequently mutated genes. Furthermore, this is a single-center study conducted in GuangDong province, which may introduce patient selection bias by only including patients in Southern China. Further research is needed to verify our findings in a multi-center study throughout China with a larger cohort.

In conclusion, this study revealed the distinct mutational features of various molecular subtypes of early-stage breast cancer among Chinese patients. Our study has identified mutational features and genomic signatures that can be used to inform therapeutic decisions or explored as potential therapeutic targets in our population, which raises the value of next-generation sequencing-based mutational analysis in clinical practice. Moreover, our study provided new insights and a deeper understanding of the differences in clinical and mutational features between Chinese and Western early-stage breast cancers.

## Data Availability Statement

The datasets presented in this study can be found in online repositories. The names of the repository/repositories and accession number(s) can be found below: National Omics Data Encyclopedia (NODE), accession number OEP001295, https://www.biosino.org/node/project/detail/OEP001295.

## Ethics Statement

The studies involving human participants were reviewed and approved by The Institutional Review Board of GDPH. The patients/participants provided their written informed consent to participate in this study.

## Author Contributions

Conceptualization: NL and WX. Data curation: WX, GZ, and BC. Formal analysis: WX, GZ, and BC. Funding acquisition: NL. Investigation: all authors. Methodology: WX, GZ, BC, ML, HL, JLiu, and HH-Z. Project administration: NL and WK. Resources: WX, GZ, and BC. Software: WX and ML. Supervision: NL and WX. EAR validation: WX, GZ, ML, and BC. Visualization: WX, GZ, ML, and BC. Roles/writing—original draft: WX, GZ, and BC. Writing—review and editing: all authors. All authors contributed to the article and approved the submitted version.

## Funding

This work was supported by funds from the National Natural Science Foundation of China (grant number: 82003066), Guangdong Provincial People’s Hospital Science and Technology Special Fund (Ph.D. Startup Project), and Guangdong Medical Science and Technology Research Fund (grant number: A2021080).

## Conflict of Interest

ML, HL, JLiu, HH-Z, and AL are employees of Burning Rock Biotech.

The remaining authors declare that the research was conducted in the absence of any commercial or financial relationships that could be construed as a potential conflict of interest.

## Supplementary Material

Supplementary Table 1 The 520 cancer-related genes included in the OncoScreen Plus panel.

Supplementary Table 2 The clinical and pathological characteristics of the 589 patients in our cohort (GDPH).

Supplementary Figure 1 Comparison of somatic genomic alterations in younger (≤35 years) patients with breast cancer in our cohort (GDPH) and the TCGA cohort. Different colors indicate different types of mutations and different clinicopathological features. Indel, insertion/deletion mutation; CN_amp, copy number amplification; CN_del, copy number deletion.

Supplementary Figure 2 Comparison of somatic genomic alterations in triple-negative breast cancer between Chinese patients (GDPH cohort) and the TCGA cohort. Different colors indicate different types of mutations and different clinicopathological features. Indel, insertion/deletion mutation; CN_amp, copy number amplification; CN_del, copy number deletion.

Supplementary Figure 3 Comparison of somatic genomic alterations of invasive lobular breast cancer (ILBC) between Chinese patients (GDPH cohort) and the TCGA cohort. Different colors indicate different types of mutations and different clinicopathological features. Indel, insertion/deletion mutation; CN_amp, copy number amplification; CN_del, copy number deletion.
