# Supplementary material for: Characterization of Frequently Mutated Cancer Genes and Tumor Mutation Burden in Chinese Breast Cancer
Source: Front Oncol. 2021 Apr 21;11:618767. doi: 10.3389/fonc.2021.618767 (PMC8096980; doi:10.3389/fonc.2021.618767)
Supplement: Supplementary Table 1 — The 520 cancer-related genes included in the OncoScreen Plus panel. [file Table_1.pdf]

Supplementary Table 1. The 520 cancer-related genes included in the OncoScreen Plus panel (Burning Rock Biotech)

ABL1	*BCL2L1*	*CDKN2B*	*EGFL7*	*FAS*	*HIST1H3B*	*IRS1*	*MDM2*	*NOTCH4*	*PIK3R3*	*RAD54L*	*SMAD4*	*TMEM127*
ABL2	*BCL2L11*	*CDKN2C*	*EGFR*	*FAT1*	*HIST1H3C*	*IRS2*	*MDM4*	*NPM1*	*PIM1*	*RAF1*	*SMARCA4*	*TMPR552*
ACVR1	*BCL2L2*	*CEBPA*	*EIF1AX*	*FAT3*	*HIST1H3D*	*JAK1*	*MED12*	*NRAS*	*PLCG2*	*RANBP2*	*SMARCB1*	*TNFAIP3*
ACVR1B	*BCL6*	*CENPA*	*EIF4A2*	*FBXW7*	*HIST1H3E*	*JAK2*	*MEF2B*	*NR4A3*	*PLK2*	*RARA*	*SMARCD1*	*TNFRSF14*
ADGRA2	*BCOR*	*CHD1*	*EIF4E*	*FCGR2B*	*HIST1H3F*	*JAK3*	*MEN1*	*NRG1*	*PMAIP1*	*RASA1*	*SMO*	*TNFSF11*
AKT1	*BCORL1*	*CHD2*	*ELOC*	*FGF10*	*HIST1H3G*	*JUN*	*MET*	*NSD1*	*PMS1*	*RB1*	*SNCAIP*	*TOP1*
AKT2	*BCR*	*CHD4*	*EMSY*	*FGF12*	*HIST1H3H*	*KAT5A*	*MGA*	*NTHL1*	*PMS2*	*RBM10*	*SOCS1*	*TOP2A*
AKT3	*BLM*	*CHEK1*	*EP300*	*FGF23*	*HIST1H3I*	*KDM5A*	*MITF*	*NTRK1*	*PNRC1*	*RECQL4*	*SOX2*	*TP53*
ALK	*BMPR1A*	*CHEK2*	*EPCAM*	*FGF6*	*HIST1H3J*	*KDM5C*	*MLH1*	*NTRK2*	*POLD1*	*REL*	*SOX9*	*TRAF2*
ALOX12B	*BRAF*	*CHUK*	*EPHA2*	*FGF7*	*HIST2H3C*	*KDM6A*	*MLH3*	*NTRK3*	*POLE*	*RET*	*SOX10*	*TRAF7*
AMER1	*BRCA1*	*CIC*	*EPHA3*	*FGFR14*	*HIST2H3D*	*KDR*	*MPL*	*NUP93*	*POM121L12*	*RFWD2*	*SOX17*	*TRRAP*
ANKRD11	*BRCA2*	*CRBN*	*EPHA5*	*FGFR1*	*HIST3H3*	*KEAP1*	*MRE11A*	*PAK1*	*PPM1D*	*RHEB*	*SPEN*	*TSC1*
APC	*BRD4*	*CREBBP*	*EPHA7*	*FGFR2*	*HLA-A*	*KEL*	*MSH2*	*PAK3*	*PPP2R1A*	*RHOA*	*SPOP*	*TSC2*
APCDD1	*BRIP1*	*CRKL*	*EPHB1*	*FGFR3*	*HNF1A*	*KIT*	*MSH3*	*PAK7*	*PPP2R2A*	*RICTOR*	*SPTA1*	*TSHR*
AR	*BTG1*	*CRLF2*	*ERBB2*	*FOXA1*	*HNF1B*	*KLF4*	*MSH6*	*PALB2*	*PPP6C*	*RIT1*	*SRC*	*U2AF1*
ARAF	*BTK*	*CSF1R*	*ERBB3*	*FOXL2*	*HOXB13*	*KLHL6*	*MST1*	*PARK2*	*PRDM1*	*RNF43*	*SRSF2*	*VEGFA*
ARFRP1	*CALR*	*CSF3R*	*ERBB4*	*FRS2*	*HRAS*	*KMT2A*	*MST1R*	*PARP1*	*PREX2*	*ROS1*	*STAG2*	*VEGFB*
ARID1A	*CARD11*	*CTCF*	*ERBB5*	*FYN*	*HSD3B1*	*KMT2C*	*MTOR*	*PARP2*	*PRKAR1A*	*RPA1*	*STAT3*	*VEGFC*
ARID1B	*CASP8*	*CTLA4*	*ERCC1*	*GABRA6*	*HSP90AA1*	*KMT2D*	*MUTYH*	*PARP3*	*PRKC1*	*RPS6KA4*	*STAT4*	*VHL*
ARID2	*CBFB*	*CTNNA1*	*ERCC2*	*GATA4*	*ICOSLG*	*KRAS*	*MYC*	*PARP4*	*PRKDC*	*RPS6KB2*	*STAT5A*	*VTCN1*
ARID5B	*CBL*	*CTNNB1*	*ERCC3*	*GATA6*	*ID3*	*LATS1*	*MYCL*	*PAX5*	*PRSS8*	*RPTOR*	*STAT5B*	*WISP3*
ASXL1	*CCND1*	*CUL3*	*ERCC4*	*GID4*	*IDH1*	*LATS2*	*MYCN*	*PBRM1*	*PTCH1*	*RUNX1*	*STK11*	*WRN*
ASXL2	*CCND2*	*CUL4A*	*ERCC5*	*GNA13*	*IDH2*	*LMO1*	*MYD88*	*PDCD1*	*PTEN*	*RUNX1T1*	*STK40*	*WT1*
ATF1	*CCND3*	*CUL4B*	*ERG*	*GPS2*	*IFNGR1*	*LRP1B*	*MYOD1*	*PDCD1LG2*	*PTK2*	*RYBP*	*SUFU*	*XIAP*
ATM	*CCNE1*	*CXCR4*	*ERRFI1*	*GREM1*	*IGF1*	*LYN*	*NBN*	*PDFRA*	*PTPN11*	*SDHA*	*SUZ12*	*XPO1*
ATR	*CD274*	*CYCLD*	*ESR2*	*GRM3*	*IGF1R*	*LZTR1*	*NCOA3*	*PDGFRB*	*PTPRD*	*SDHAF2*	*SYK*	*XRCC2*
ATRX	*CD276*	*CYP17A1*	*EWSR1*	*GSK3B*	*IGF2*	*MAG12*	*NCOR1*	*PDK1*	*PTPRS*	*SDHB*	*TACC3*	*XRCC3*
AURKA	*CD79A*	*DAXX*	*EZH2*	*GSTM1*	*IKBKE*	*MALT1*	*NEB*	*PGR*	*PTPRT*	*SDHC*	*TAF1*	*YAP1*
AURKB	*CD79B*	*DCUN1D1*	*FAM175A*	*GSTT1*	*IKZF1*	*MAP2K1*	*NEGR1*	*PHOX2B*	*QK1*	*SDHD*	*TBX3*	*YES1*
AXIN1	*CDC73*	*DDR2*	*FAM46C*	*H3F3A*	*IL10*	*MAP2K2*	*NF1*	*PIK3CA*	*RAB35*	*SETD2*	*TCF3*	*ZBTB2*
AXIN2	*CDH1*	*DICER1*	*FANCA*	*H3F3B*	*IL7R*	*MAP2K4*	*NF2*	*PIK3CB*	*RAC1*	*SF3B1*	*TCF7L2*	*ZFHX3*
AXL	*CDK12*	*DIS3*	*FANCC*	*HDAC1*	*INHA*	*MAP3K1*	*NFE2L2*	*PIK3C2B*	*RAD21*	*SH2B3*	*TERC*	*ZNF217*
B2M	*CDK4*	*DNAJB1*	*FANCD2*	*HDAC2*	*INHBA*	*MAP3K13*	*NFKB1A*	*PIK3C2G*	*RAD50*	*SH2D1A*	*TERT*	*ZNF703*
BACH1	*CDK6*	*DNMT1*	*FANCE*	*HDAC4*	*INPP4A*	*MAP3K14*	*NKX2-1*	*PIK3C3*	*RAD51*	*SHQ1*	*TET1*	*ZNRF3*
BAP1	*CDK8*	*DNMT3A*	*FANCF*	*HGF*	*INPP4B*	*MAP3K3*	*NKX3-1*	*PIK3CD*	*RAD51B*	*SLIT2*	*TET2*	*ZRSR2*
BARD1	*CDKN1A*	*DNMT3B*	*FANCG*	*HIST1H1C*	*INSR*	*MAPK1*	*NOTCH1*	*PIK3CG*	*RAD51C*	*SLX4*	*TGFBR1*	
BBC3	*CDKN1B*	*DOT1L*	*FANCI*	*HIST1H2BD*	*IRF2*	*MAX*	*NOTCH2*	*PIK3R1*	*RAD51D*	*SMAD2*	*TGFBR2*	
BCL2	*CDKN1C*	*E2F3*	*FANCL*	*HIST1H3A*	*IRF4*	*MCL1*	*NOTCH3*	*PIK3R2*	*RAD52*	*SMAD3*	*TIPARP*	
BCL10	*CDKN2A*	*EED*	*FANCM*									

Supplementary Table 2. The clinical and pathological characteristics of the 589 patients in our cohort (GDPH)

Characteristics		No.(%)
Age (years)	(median, min~max)	(48, 22~85)
	>50	241(40.9%)
	≤50	348(59.1%)
HR/HER2 status	HR-/HER2+	64(10.9%)
	HR+/HER2+	111(18.8%)
	HR+/HER2-	321(54.5%)
	TNBC	68(11.5%)
	Unknown	25(4.2%)
Molecular subtypes	Luminal A	114(19.4%)
	Luminal B	
	Luminal B/HER2-negative	207(35.1%)
	Luminal B/HER2-positive	111(18.8%)
	HER2-enriched	64(10.9%)
	TNBC	68(11.5%)
	Unknown	25(4.2%)
Histological type	IDC	514(87.3%)
	ILC	14(2.4%)
	OTHER	61(10.4%)
Pathological stage	I	137(23.3%)
	II	329(55.9%)
	III	123(20.9%)
Pathologic T status	T1	228(38.7%)
	T2	327(55.5%)
	T3	24(4.1%)
	T4	10(1.7%)
Pathologic N status	N0	253(43.0%)
	N1	220(37.4%)
	N2	82(13.9%)
	N3	34(5.8%)
	Unknown	0
Pathologic M status	M0	589(100.0%)
	M1	0
	Unknown	0
Menopausal status	Pre-menopausal	336(57.0%)
	Post-menopausal	253(43.0%)
Histological grade	I	22(3.7%)
	II	274(46.5%)
	III	282(47.9%)
	Unknown	11(1.9%)

Abbreviations: HR, hormone receptor; HER2, human epidermal growth factor receptor 2; TNBC, triple-negative breast cancer; IDC, invasive ductal carcinoma; ILC, invasive lobular carcinoma; T, tumor status; N, nodal status; M, metastatic status
