# Supplementary material for: Characterization of Frequently Mutated Cancer Genes and Tumor Mutation Burden in Chinese Breast Cancer
Source: Front Oncol. 2021 Apr 21;11:618767. doi: 10.3389/fonc.2021.618767 (PMC8096980; doi:10.3389/fonc.2021.618767)
Supplement: Supplementary Figure 1 — Comparison of somatic genomic alterations in younger (≤35 years) patients with breast cancer in our cohort (GDPH) and the TCGA cohort. Different colors indicate different types of mutations and different clinicopathological features. Indel, insertion/deletion mutation; CN_amp, copy number amplification; CN_del, copy number deletion. [file Image_1.pdf]

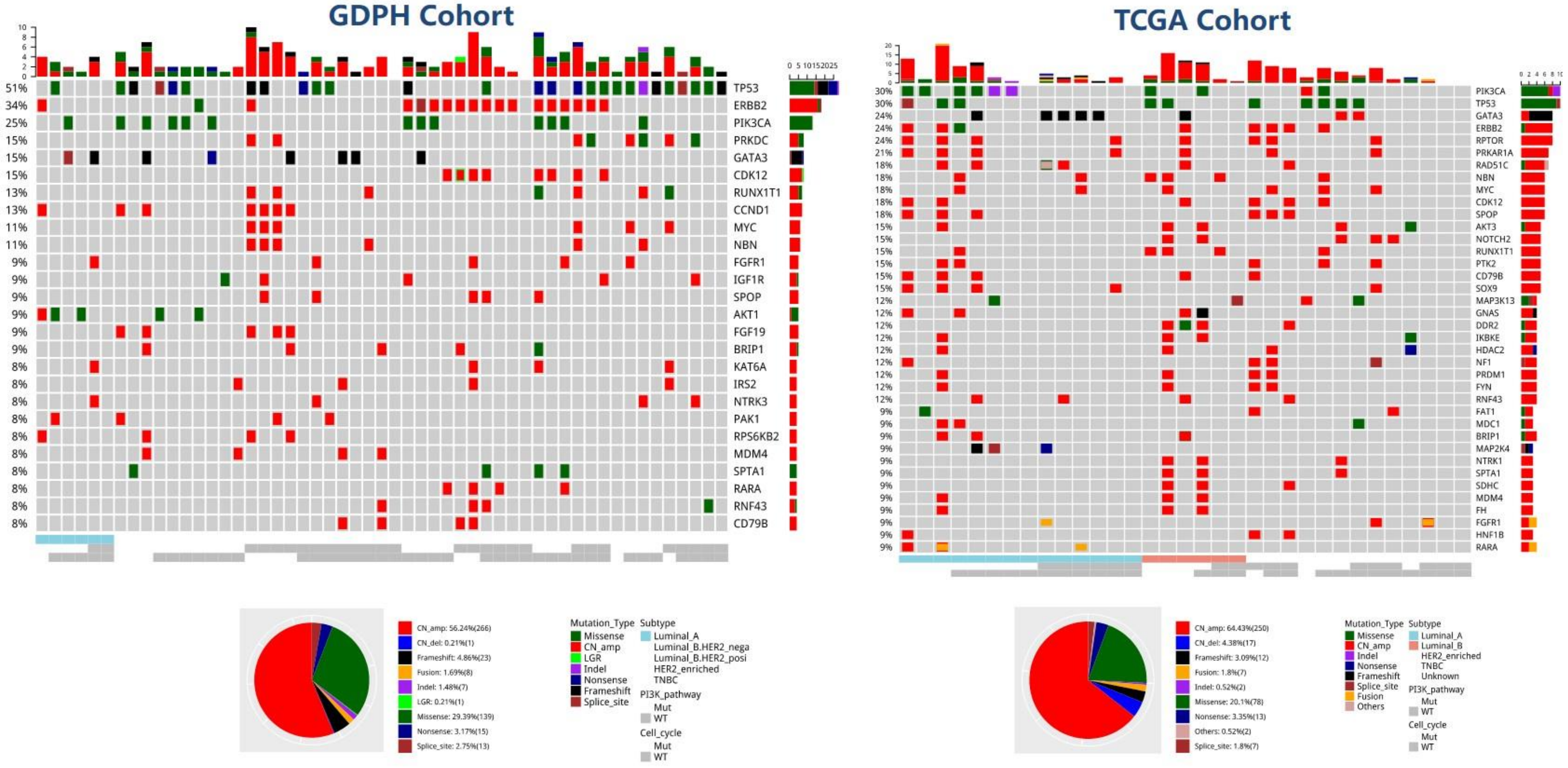

Supplementary Figure 1. Comparison of somatic genomic alterations in younger (≤35 years) patients with breast cancer in our cohort (GDPH) and the TCGA cohort. Different colors indicate different types of mutations and different clinicopathological features. Indel, insertion/deletion mutation; CN_amp, copy number amplification; CN_del, copy number deletion.

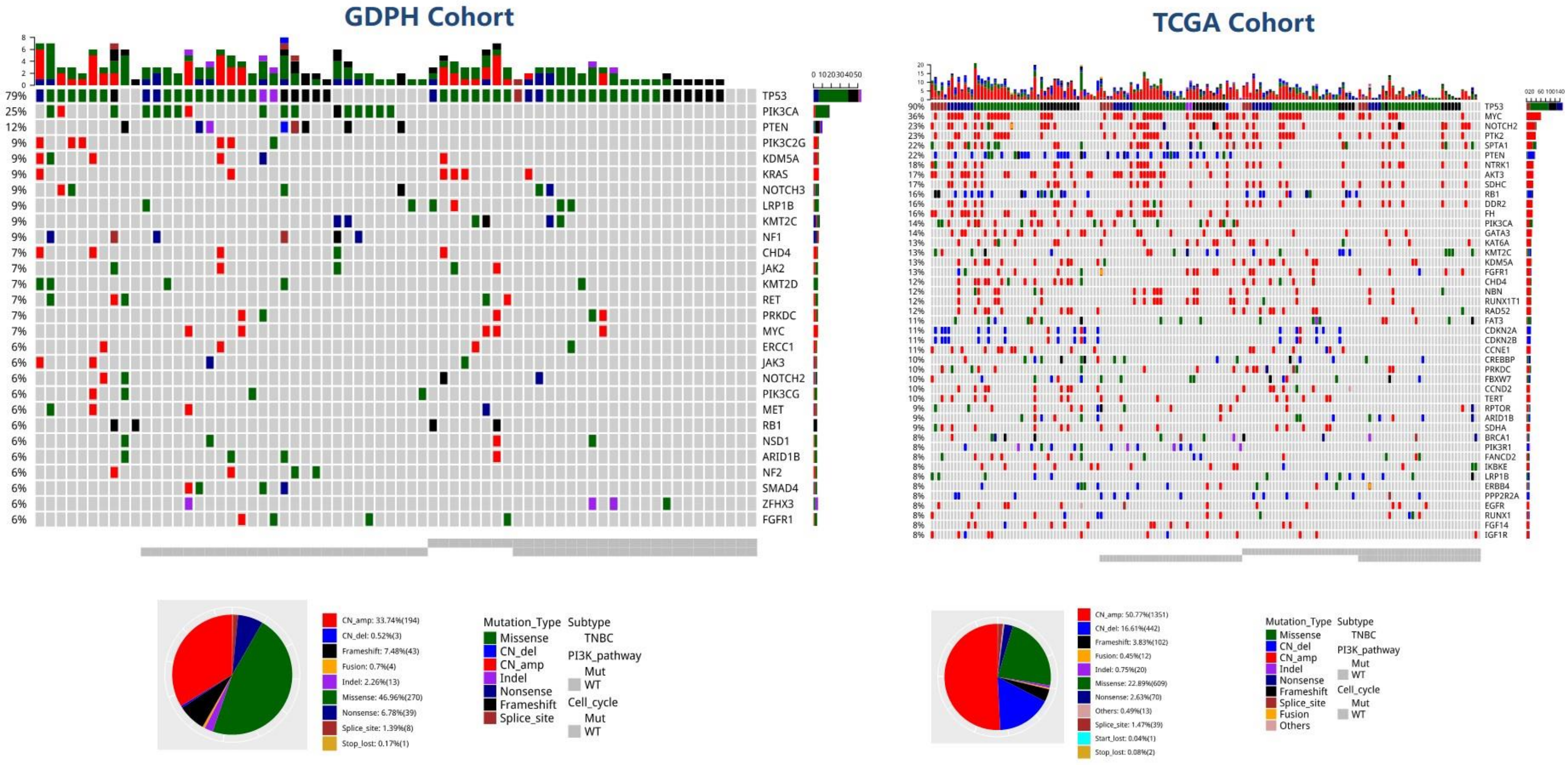

Supplementary Figure 2. Comparison of somatic genomic alterations in triple-negative breast cancer between Chinese patients (GDPH cohort) and the TCGA cohort. Different colors indicate different types of mutations and different clinicopathological features. Indel, insertion/deletion mutation; CN_amp, copy number amplification; CN_del, copy number deletion.

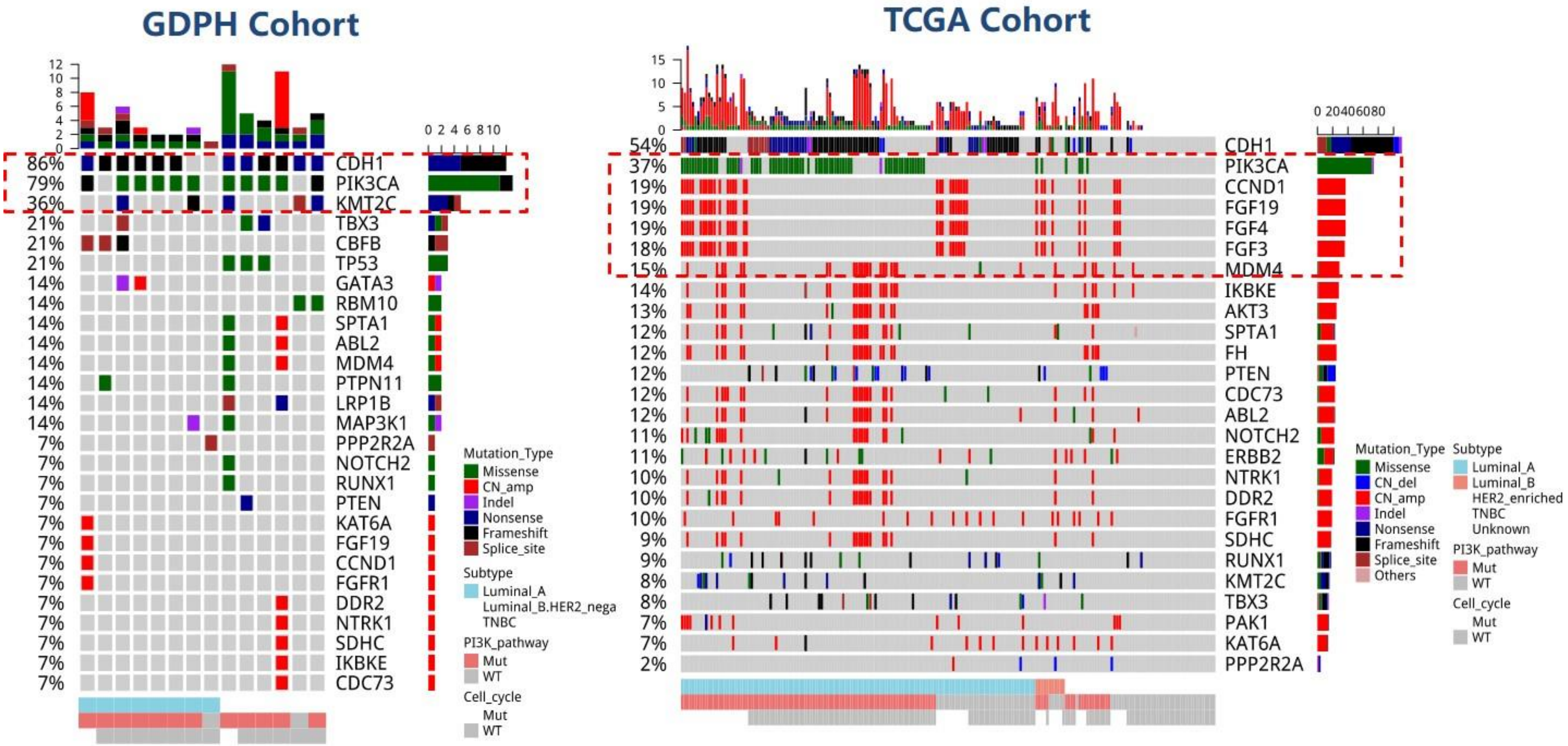

Supplementary Figure 3. Comparison of somatic genomic alterations of invasive lobular breast cancer (ILBC) between Chinese patients (GDPH cohort) and the TCGA cohort. Different colors indicate different types of mutations and different clinicopathological features. Indel, insertion/deletion mutation; CN_amp, copy number amplification; CN_del, copy number deletion.
